# Making blind spots visible: the lack of race-conscious research and data in public and global health

**DOI:** 10.1093/eurpub/ckac131.523

**Published:** 2022-10-25

**Authors:** M Meudec, C Affun-Adegbulu, T Cosaert, T Verdonck

**Affiliations:** 1 Department of Public Health, Institute of Tropical Medicine, Antwerpen, Belgium; 2 Outbreak Research Team, Institute of Tropical Medicine, Antwerpen, Belgium; 3 Data Science Hub, Institute of Tropical Medicine, Antwerpen, Belgium

## Abstract

In Continental Europe, race-conscious research and data (RCRD) is not adequately deployed in support of policy-making and social change to tackle health disparities. One reason is a lack of national data systems based on race in many countries, another is that researchers or policymakers are unaccustomed to taking them into account. One consequence of this for public and global health is the adoption and implementation of policies that are ostensibly race-neutral but which in fact reproduce “methodological whiteness” (Bhambra, 2017) and create or exacerbate racial health inequities. Calls to decolonise public health recognize the importance of RCRD, yet race or ethnicity as a variable in research continues to be seen as a contentious issue for a number of reasons. First, while some researchers see it as a valuable tool for addressing health inequalities or the impact of institutionalized and systemic racism on racialized/ethnic minority groups, others oppose its use on the ground that it can lead to stigmatization or racial stereotyping. Second, RCRD is often seen as too difficult to implement in practice, due to challenges of finding appropriate approaches to conceptualisation, operationalisation, data (non)collection, and interpretation. Third, many researchers fear that the use of race and ethnicity in research may be instrumentalized against minorities in ways that are sometimes difficult to anticipate. This project aims to contribute to addressing this, by building expertise for RCRD and creating a knowledge network and community of practice for researchers working in Public and Global Health in Continental Europe. The project begins by examining how RCRD is used at the Institute of Tropical Medicine Antwerp, which is located in Belgium, Europe but has research collaborations on other continents. It then continues with a scoping review on the use of RCRD to address racial and ethnic health disparities more generally across Europe.

**Key messages:**

• Race-conscious research and data are lacking in public and global health research and policy-making, leading to racial health disparities.

• Need to develop expertise and a network of researchers in race-conscious research and data in Europe.

